# Integrating scRNA-seq and GWAS data reveals potentially critical endothelial cells in large artery atherosclerotic stroke

**DOI:** 10.3389/fnins.2025.1646993

**Published:** 2025-09-02

**Authors:** Hong Deng, Qiu-Xue Weng, Mei Zeng, Yan Li, Qi Pan

**Affiliations:** Department of Neurology, Affiliated Hospital of Guizhou Medical University, Guiyang, China

**Keywords:** stroke, endothelial cells, scRNA-seq, GWAS, S-LDSC

## Abstract

**Introduction:**

Stroke is a devastating cerebrovascular disease with limited treatment options. Structural and functional abnormalities in the cerebral vasculature contribute significantly to the development of stroke. Specifically, the blood–brain barrier formed by a combination of endothelial cells, smooth muscle cells, pericytes, and glial cells is proposed to play a critical role in stroke pathogenesis. However, the primary causative cell types in stroke are not well-defined.

**Methods:**

Here we integrated single-cell RNA-sequencing data from cerebrovascular zonation structures with genome-wide association study (GWAS) data of various types of strokes to analyze stroke risk SNP loci enrichment in various cell types through heritability partitioning. Fourteen vascular cell clusters were identified and profiled using cell-type expression-specific integration for complex traits (CELLEX) to assess gene expression specificity. Heritability enrichment was analyzed across three GWAS datasets: general stroke, large-artery atherosclerotic stroke (Stroke_Large_AS), and small-vessel ischemic stroke. Gene set enrichment analysis (GSEA) and pseudotime trajectory modeling (Monocle2) were performed to characterize the functional and developmental dynamics of critical endothelial subtypes. Transcriptional regulatory networks were further interrogated using SCENIC.

**Results:**

We found two specific endothelial cell subtypes play a potentially critical role in large artery atherosclerotic stroke. These subtypes demonstrated distinct expression profiles and pathway enrichments, with aEC_cluster associated with atherosclerosis and inflammatory signaling, and EC2_cluster with epithelial cell regulation and muscle tissue development. Pseudotime analysis revealed that EC2_cluster cells represent an earlier endothelial state that differentiates into the aEC_cluster, the terminal pathogenic state. Gene regulatory network analysis identified transcription factors Gata2, Irf7, and Jund as central regulators in aEC_cluster differentiation, orchestrating the upregulation of key molecules such as Fos and Nr4a1.

**Conclusion:**

Our study provides insights into the molecular mechanisms in stroke and suggests new therapeutic strategies for this debilitating disease.

## Introduction

Stroke is the leading cause of disability and the second leading cause of death globally (GBD 2019 Stroke Collaborators, 2021). Over 80% of strokes are ischemic events, which can cause significant neurological, morphological, and molecular abnormalities (Shehjar et al., 2023). To date, effective treatments for ischemic stroke remain lacking (Datta et al., 2020; Wen-Juan et al., 2023). Therefore, further investigation into the causes of ischemic stroke onset is crucial to prevent its occurrence and reduce the disease burden (Beylerli et al., 2024).

The health of the brain is dependent on the health of its vasculature, with the blood–brain barrier (BBB) being a specialized brain vascular structure that supports cerebral blood flow and neurological function under normal conditions (Mei-Yun et al., 2022; Profaci et al., 2020). Comprising various cell types, including endothelial cells, smooth muscle cells, perivascular cells, and glial cells, the BBB functions collectively to maintain its essential properties (Jonas et al., 2024; Veltkamp and Gill, 2016). Abnormalities within the BBB components, such as damage to vascular endothelial cells, can result in thrombosis and subsequent blockage of blood vessels, ultimately leading to the development of ischemic stroke (Edwards and Bix, 2019). Recent advancements in single-cell technology have enabled the exploration cellular heterogeneity within brain vascular structures (He et al., 2018; Pan et al., 2022; Yang et al., 2022). However, it remains unclear whether specific cell types within the brain vasculature contribute to the pathogenesis of ischemic.

Genome-wide association analysis (GWAS) has emerged as a powerful tool to identify potential causal loci for diseases by associating complex phenotypes with genotypes (Barbeira et al., 2019). Recent GWAS studies have successfully identified several risk loci for stroke (Malik et al., 2018; Kilarski et al., 2014; Holliday et al., 2012). Integration of expression data from single-cell RNA sequencing (scRNA-seq) with GWAS statistics using stratified linkage disequilibrium score regression (S-LDSC) enables identification of possible pathogenic cell types of a disease through heritability enrichment (Gareev et al., 2024). In this study, we utilized an algorithm called cell-type expression-specific integration for complex traits (CELLECT) (Timshel et al., 2020) to integrate single-cell profiles of the mouse cerebral vasculature with GWAS statistics from stroke, large-artery atherosclerotic stroke, and small-vessel stroke. Our analysis revealed the presence of two specific endothelial cell types, namely aEC_cluster and EC2_cluster, which are potential associated with large atherosclerotic stroke. Furthermore, gene set enrichment analysis (GSEA) revealed that these two cell subtypes are specifically associated with atherosclerosis and epithelial cell proliferation, respectively, providing further evidence of their crucial role in the development of large artery atherosclerotic stroke. Although our conclusions are based solely on existing scRNA-seq and GWAS data, analysis of transcriptional regulatory networks provides potential targets for the prevention and treatment of stroke in clinical settings.

## Materials and methods

### Data collection

The open GWAS database^1^ was utilized to obtain GWAS statistics for different types of stroke, including ebi-a-GCST006906 for stroke, ebi-a-GCST005840 for large artery atherosclerosis stroke, and ebi-a-GCST006909 for small vessel stroke (Malik et al., 2018). To ensure adequate overlap between the genes obtained from single-cell data and the GWAS risk loci, we integrated a brain vascular single-cell atlas (Vanlandewijck et al., 2018), which was obtained using SMART-seq2-based technology. Specifically, we analyzed the brain vascular single-cell dataset from the Gene Expression Omnibus database (GEO; https://www.ncbi.nlm.nih.gov/geo/) using accession numbers GSE98816 (Vanlandewijck et al., 2018).

### Data processing of scRNA-seq data

We processed and integrated our raw single-cell count matrices using the Seurat package (version 4.0; https://satijalab.org/seurat/). To ensure data quality, we excluded low-quality cells which expressed ≤200 genes, ≥20,000 unique molecular identifiers, or ≥20% mitochondrial genes. After that, we performed normalization and dimensionality reduction using the t-Distributed Stochastic Neighbor Embedding (t-SNE) algorithm (Skrodzki et al., 2024) for non-linear dimensionality reduction. Clusters were identified and annotated using previously published data (He et al., 2018).

### Quantifying expression specificity in cell types using CELLEX

To identify a set of genes that are expressed in specific cell types and to quantify their expression specificity (ES), we used CELLEX (CELL type EXpression-specificity) (Timshel et al., 2020). This tool allowed us to measure the ES of each gene in the expression matrix of each cell type. To simplify the analysis, we calculated a single ES estimate (ESμ) for each gene based on its expression in each cell type. This score represents the degree of specificity of the gene’s expression to the cell type in which it is expressed.

### Identification of critical cell types in stroke through scRNA-seq and GWAS integration

To systematically prioritize cell types that may play etiological roles in complex diseases such as stroke, we employed an integrative analytical framework that combines scRNA-seq data with GWAS summary statistics. Specifically, we utilized the CELLECT tool (Timshel et al., 2020), which incorporates gene expression specificity information across cell types to evaluate heritability enrichment using S-LDSC. The CELLECT workflow consists of the following key steps: (1) Calculation of cell type-specific expression profiles: For each cell type or cluster identified in the scRNA-seq dataset, CELLECT quantifies expression specificity by computing metrics such as the mean expression and ESμ score (expression specificity metric), identifying genes that are highly specific to each cell Type. (2) Gene annotation to regulatory regions: Each cell type-specific gene is annotated to a genomic region that includes a ± 100 kilobase (kb) window surrounding the transcription start site, capturing nearby regulatory elements that may influence gene expression. (3) Linkage disequilibrium (LD) score computation: For each annotated gene region, CELLECT calculates LD scores for HapMap3 SNPs, quantifying the extent to which these variants are correlated with each other across the population. This step creates a cell type-specific annotation map that reflects the distribution of regulatory elements across the genome. (4) Heritability enrichment analysis: Finally, the framework assesses whether the SNPs associated with a given trait or disease (e.g., stroke or stroke subtypes) are significantly enriched in the gene sets specific to certain cell types. This is done through S-LDSC, which models the contribution of each annotated SNP set to the overall SNP-based heritability of the trait. A statistically significant enrichment indicates that the corresponding cell type may be critically involved in the pathogenesis of the disease. By integrating GWAS summary statistics for stroke and its subtypes with high-resolution scRNA-seq data from cerebrovascular cells, this approach enables the identification of cell types whose gene expression landscapes are disproportionately enriched for genetic risk variants, thereby pinpointing potential cellular origins of disease susceptibility.

### Differential expression analysis and functional enrichment analysis

To identify differentially expressed genes between cell subpopulations, we utilized the “Findmarker” function in the Seurat package. GSEA was performed using the “clusterProfiler” package.

### Pseudotime-trajectory analysis

We analyzed the developmental trajectory of cell types using the Monocle2 (v2.12.0) R package (Trapnell et al., 2014; Qiu et al., 2017). To identify gene ordering (qval <0.01), we utilized the differential GeneTest function of the Monocle2 package. Subsequently, we utilized the reduce Dimension function and performed the order Cells function with default parameters to infer the trajectory.

### Network analysis for transcriptional regulators

pySCIENIC (Single-Cell rEgulatory Network Inference and Clustering)(version 0.11.0) was used to identify single-cell gene regulatory network (GRN) (Van de Sande et al., 2020). To construct the GRNs, we first generated an expression matrix and co-expression modules of transcription factors (TFs) and their potential target genes based on co-expression analysis. We then performed TF motif analysis using RcisTarget to identify direct-binding regulons. To evaluate the activity of the regulons in each cell, we used AUCell (regulon_AUC) to calculate the regulon activity score (RAS), which represents the average activity score across regulons. Finally, we summarized the GRNs and regulons for the endothelial cell types of mouse cerebrovascular cells.

## Results

### Data integration and profiling of single-cell data

**Figure 1:**
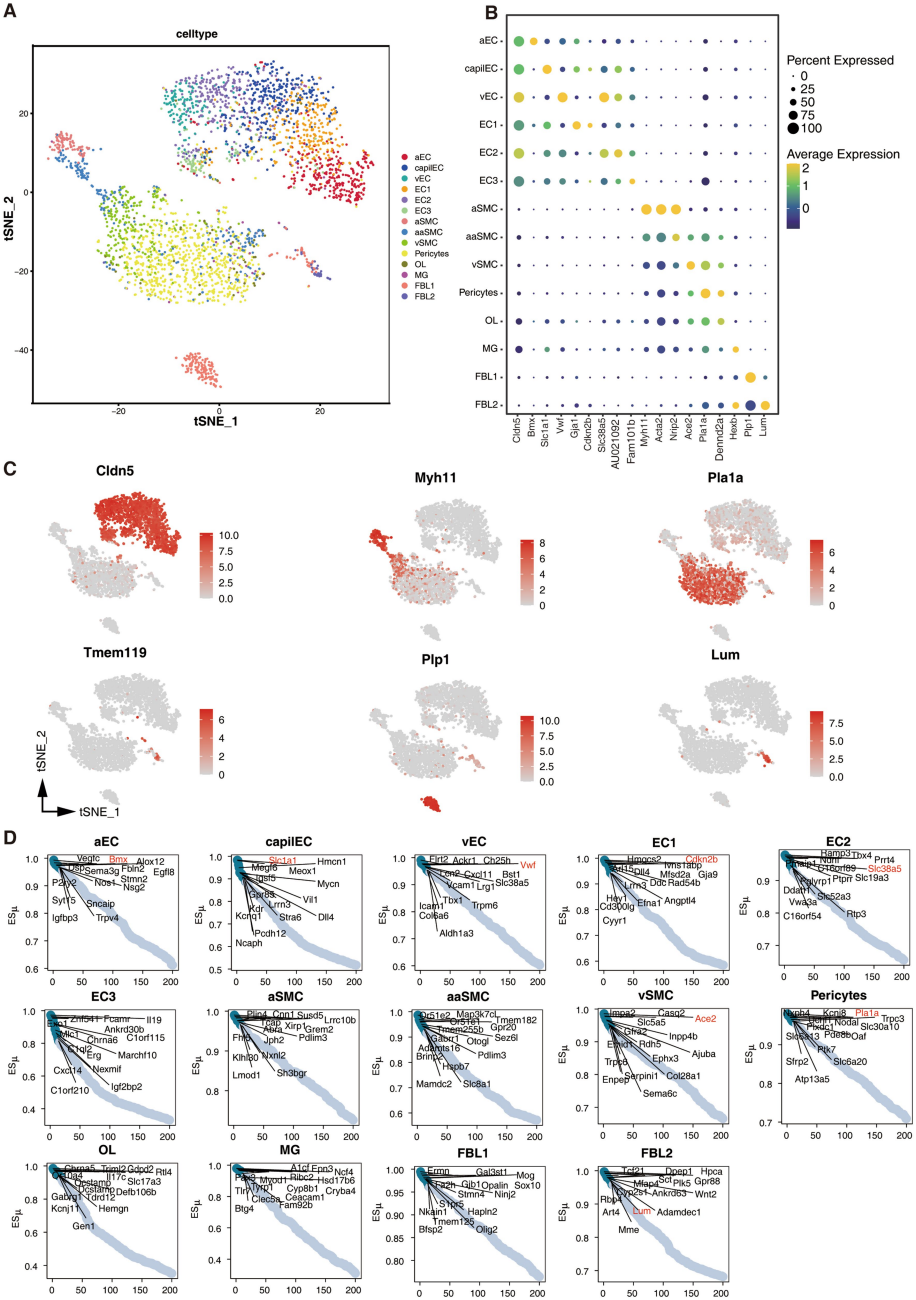
Single-cell landscape of brain vasculature and cell subtype-specific gene expression. **(A)** t-Distributed stochastic neighbor embedding (t-SNE) plot showing the cell types of brain vasculature represented by diverse colors: aEC (arterial endothelial cell), capilEC (capillary endothelial cell), vEC (venous endothelial cell), EC1, EC2, EC3, aSMC (arterial smooth muscle cell), aaSMC (arteriolar smooth muscle cell), vSMC (venous smooth muscle cell), Pericytes, OL (oligodendrocyte-lineage cells), MG (Microglia), FBL1 (vascular Fibroblast-like cell 1), FBL2 (Vascular Fibroblast-like cell 2). **(B)** Dot plot showing the percentage of featured genes expressed in each cluster and their average expression levels. **(C)** Feature plots demonstrating the expression of selective marker genes of cerebrovascular cells. **(D)** Scatter plots showing the ordering of specific genes expressed in each cluster obtained by the CELLEX algorithm.

We conducted a comprehensive analysis of a scRNA-seq dataset obtained from a recent study (He et al., 2018), which aimed to uncover the gene expression profiles of vascular and vessel-associated cells across defined cerebrovascular zonation in the mouse brain. Our analysis identified 14 distinct cell clusters using t-Distributed stochastic neighbor embedding (Figure 1A), and revealed the top genes that were specifically expressed in each cell cluster (Figure 1B). Notably, genes such as Cldn5, Myh11, Pla1a, Theme119, Plp1, and Lum, were consistently expressed at higher levels in their respective cell clusters (Figure 1C).

To quantitatively assess and rank gene expression specificity in the 14 cerebrovascular cell subtypes, we utilized the CELLEX toolkit. The ESμ score was utilized to represent the degree of gene expression specificity in each cell cluster. Our analysis identified the most specifically expressed genes in each cell cluster (Figure 1D), and their expression patterns were consistent with those observed in the dot plot of average expression. These findings indirectly validated the reliability of the ESμ score in assessing gene expression specificity.

### Two endothelial cell subtypes are specifically associated with risk loci for large artery atherosclerotic stroke

**Figure 2:**
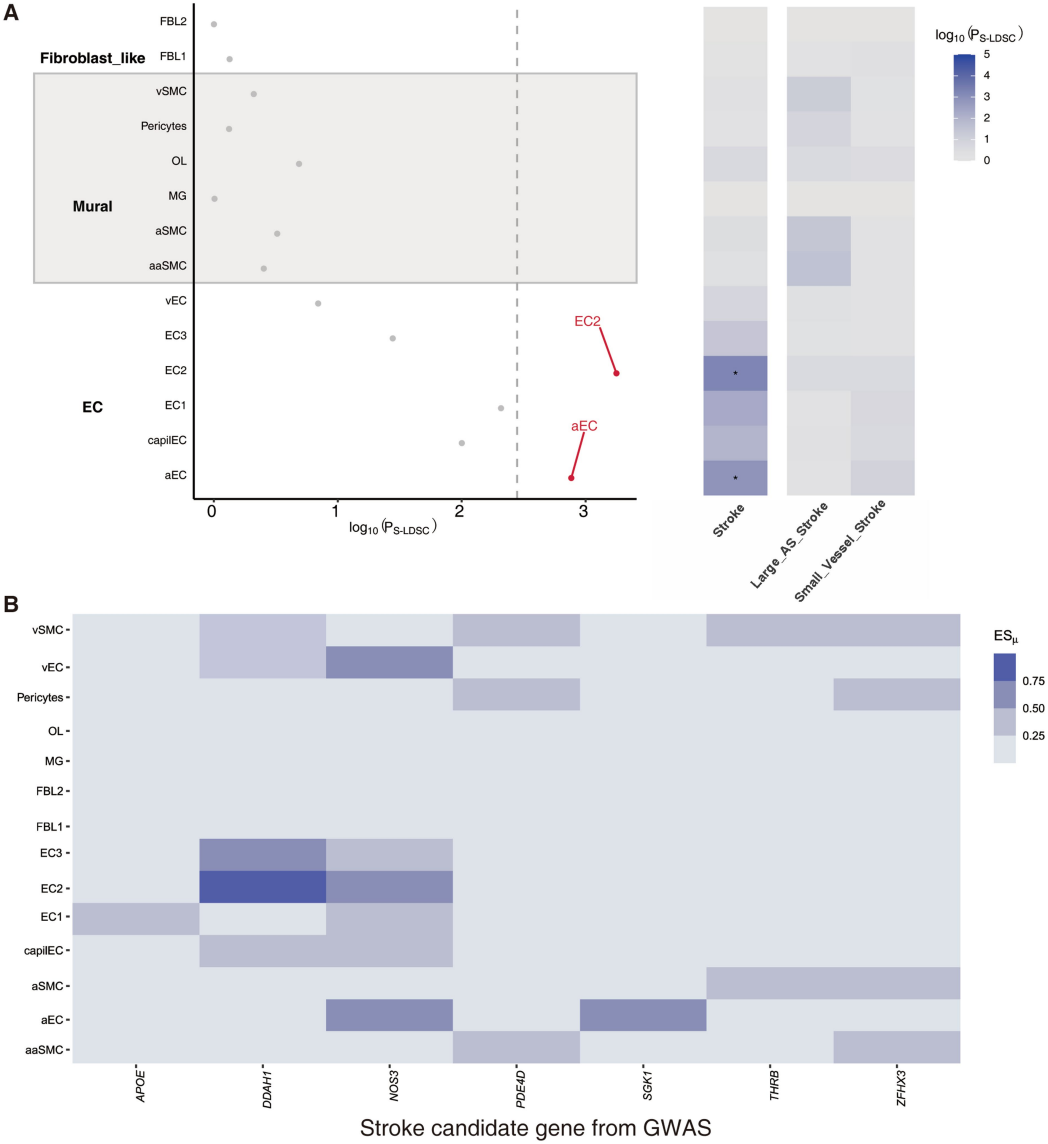
Two endothelial cell subtypes are significantly enriched at risk loci for large artery atherosclerotic stroke. **(A)** Prioritization of the 14 mouse cerebrovascular cell subtypes identified two endothelial cell subtypes that were significantly associated with atherosclerotic stroke, which are highlighted in red and have passed the Bonferroni significance threshold, P_S-LDSC_ <0.05/14. **(B)** Heatmap showing the expression levels of the stroke candidate genes at stroke risk loci in different vascular cell subtypes. AS, Atherosclerotic.

To identify vascular cell types associated with stroke, we employed the CELLECT algorithm to analyze the 14 vascular cell types using three different GWAS statistics, namely stroke, Stroke_Large_AS, and ischemic Stroke_small_vessel. Our analysis revealed that two of the EC clusters, namely the aEC cluster and EC2 cluster, showed significant enrichment for variants associated with Stroke_large_AS (Figure 2A). Moreover, we assessed the ESμof seven stroke candidate genes obtained from a GWAS study (Bevan et al., 2012), which supported the reliability of our findings (Figure 2B). Notably, several of these candidate genes, including DDAH1, NOS3, and SGK1, are specifically enriched in either the aEC_cluster or the EC2_cluster, providing insights into the molecular mechanisms by which these cell types contribute to the pathogenesis of large artery atherosclerotic stroke.

### Enrichment of atherosclerosis-related pathways in the two potentially critical endothelial cell subtypes

**Figure 3:**
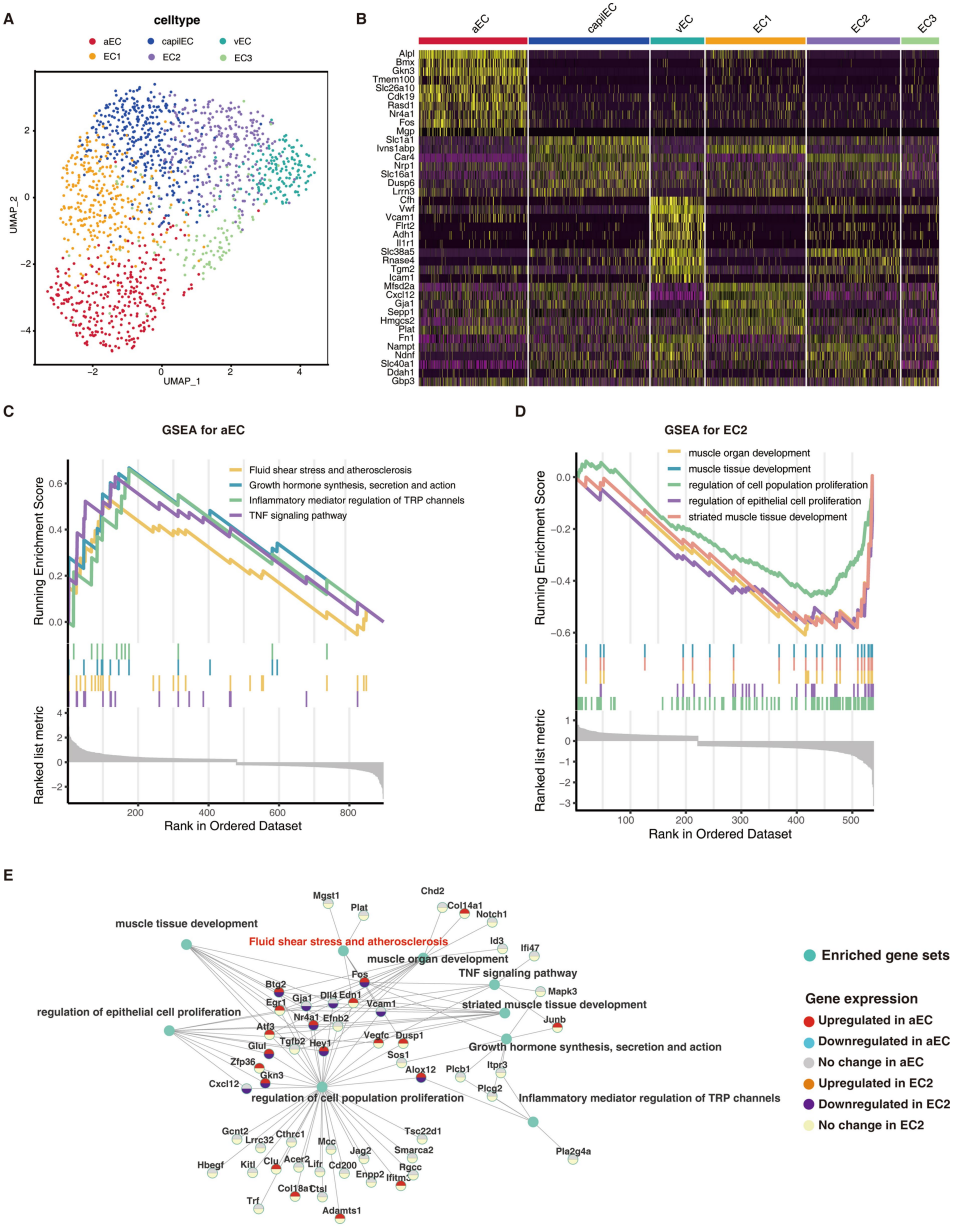
Critical endothelial cell subtypes associated with atherosclerosis. **(A)** Uniform manifold approximation and projection (UMAP) plot for endothelial cell subtypes. **(B)** Heatmap showing the expression of marker genes in the six different cell subtypes with the color of the heat map indicating the level of expression. **(C,D)** Significantly up-regulated pathways in aEC and down-regulated pathways in EC2 obtained by GSEA enrichment analysis. **(E)** Grid diagram showing enriched gene sets and their respective representative/associated genes significantly enriched in aEC_cluster or EC2_cluster. The colors of the dots indicate change directions of the genes in the two cell subtypes.

To explore the relationship between stroke pathology and the two distinct subtypes of endothelial cells, we employed GSEA to analyze their cellular functions (Figures 3A–D). Our findings revealed significant enrichment of specific pathways in each subtype. In the aEC_cluster, we identified a significant upregulation of pathways associated with fluid shear stress and atherosclerosis, growth hormone synthesis, inflammatory mediator regulation of TRP channels, and TNF signaling (Figure 3C). Conversely, the EC2_cluster exhibited a significant downregulation of pathways related to muscle organ development, muscle tissue development, and regulation of epithelial cell proliferation (Figure 3D). These findings suggest distinct functional profiles and underlying molecular processes associated with the two critical endothelial cell subtypes.

Moreover, we identified several key molecules, including Nr4a1, Fos, and Alox12, that exhibited significant changes in both aEC_cluster and EC2_cluster (Figure 3E). These molecules are known to be involved in crucial biological processes, highlighting their potential roles in mediating the pathogenesis of stroke within these endothelial cell subtypes.

### The two potentially critical endothelial cell subtypes are at distinct stages along the same differentiation trajectory

**Figure 4:**
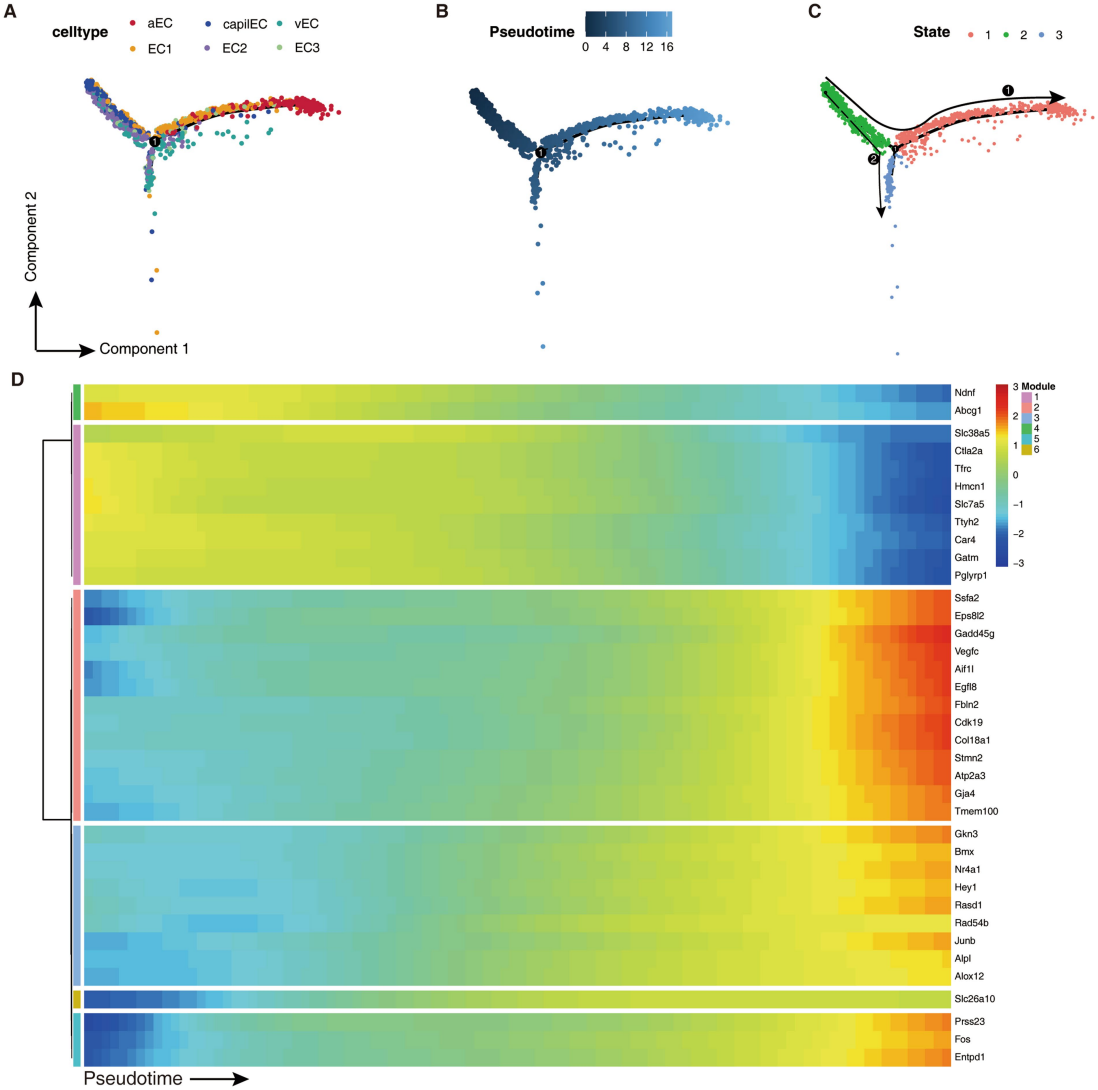
Developmental trajectory of endothelial cells. **(A–C)** Distribution of cell types, pseudotime and states along the trajectory of cell differentiation. **(D)** Heatmap showing expression level changes of various genes over pseudotime, with color of the legend indicating the level of gene expression. The colored modules on the left side of the heatmap indicate the six expression modules obtained by unsupervised clustering of gene expression changes.

To gain insights into the differentiation trajectory of the two stroke-associated endothelial cell subtypes, we performed pseudotime analysis on all endothelial cells using Monocle2. Our analysis revealed a differentiation trajectory characterized by the bifurcation of endothelial cells into two states: state 2 cells differentiating toward either state 1 or state 3. Specifically, the aEC_Cluster mainly belonged to state 1, while EC2_Cluster predominantly belonged to state 2. As a result, the two pathogenic EC classes within the EC2_Cluster gradually transitioned toward aEC_Cluster differentiation (Figures 4A–C). Notably, the aEC_Cluster represents the terminal stage of differentiation.

Furthermore, we performed unsupervised clustering of gene expression kinetic patterns and identified six expression modules shared by endothelial cells. Intriguingly, genes within modules 2, 3 and 5 showed a gradual elevation in expression as pseudotime advanced (Figure 4D). Consistent with our findings in Figure 3, differential expression and functional enrichment analysis revealed the gradual upregulation of genes associated with atherosclerosis, such as FOS, NR4A1, and alox12, along the differentiation trajectory from EC2_Cluster to aEC_Cluster.

### Key regulatory networks for endothelial cell differentiation into potentially critical subtypes

**Figure 5:**
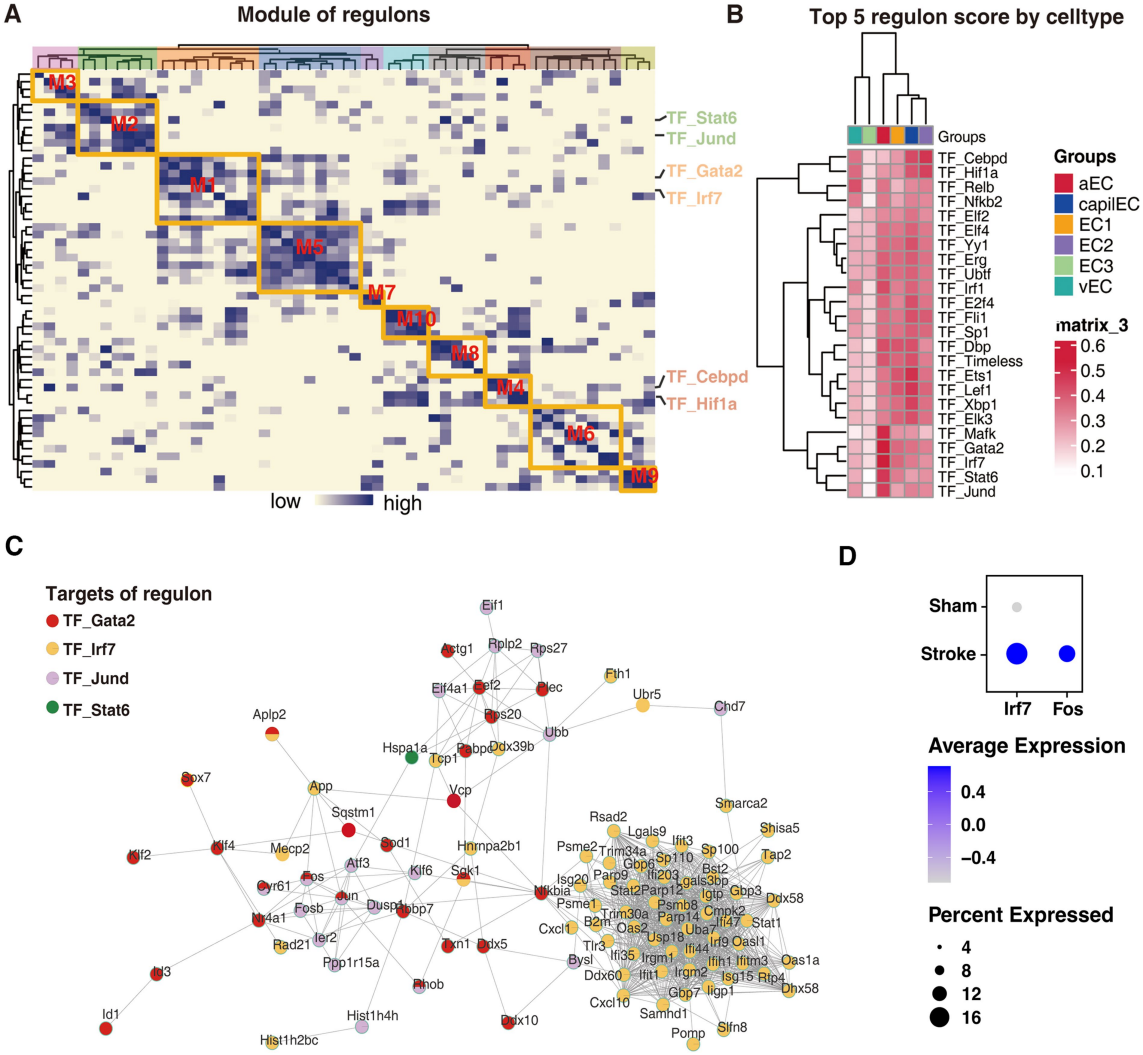
Transcriptional regulatory networks driving endothelial cell differentiation into potentially critical subtypes. **(A)** Co-expression modules of the endothelial cell transcriptional regulatory network, with the color of the heatmap indicating the correlation of the corresponding transcriptional regulators. **(B)** The activity of the top 4 transcriptional regulators for each cell subtype. **(C)** The grid showing the most active transcriptional regulators and their targets in aEC_cluster. **(D)** Incorporated scRNA-seq data showing the levels of Ifr7 and Fos in the MCAO model group were significantly higher than those in the control group.

To investigate the upstream transcriptional regulatory events that play potential key roles in the differentiation of endothelial cells into these critical cell subtypes, we conducted a gene regulatory network analysis. Using SCENIC’s integrated algorithm, we identified 10 transcriptional regulator modules of regulons (i.e., transcription factor with its target) and their co-expression modules in all the endothelial cells (Figure 5A; Supplementary Table 1). We further evaluated the scoring of each endothelial cell subtype in these modules and found that aEC_cluster had the highest scores in modules M1 and M2, while EC2_cluster had the highest score in M4 (Supplementary Figure 1). To identify the key transcriptional regulatory network in the two potentially critical cell subtypes associated with stroke, we first scored the regulons in each subtype (Supplementary Table 2). From these results, the top regulons enriched in each subtype were identified (Figure 5B). Notably, the results revealed that the activity of the top five regulons, namely TF_Mafk, TF_Gata2, TF_Irf7, TF_Stat6 and TF_Jund, was significantly elevated in aEC (Figure 5B). Further analysis revealed the extensive regulation of molecular networks in aEC by transcription factors Gata2, Irf7, and Jund. Remarkably, this regulation included molecules such as Fos and Nr4a1, which were displayed progressively elevation during aEC_cluster differentiation in the pseudotime analysis (Figures 4D, 5C). To further validate the reliability of our transcriptional regulatory factor predictions, we incorporated scRNA-seq data from an external model of middle cerebral artery occlusion (MCAO) in the brain (Zhang et al., 2023). The results indicated that the levels of Ifr7 and Fos in the MCAO model group were significantly higher than those in the control group (Figure 5D), thereby suggesting the reliability of our findings to some extent.

## Discussion

In this study, we aimed to infer the risk of stroke associated with cell types by integrating single-cell datasets of the cerebral vasculature with GWAS data from various stroke types. Using S_LDSC heritability segmentation, we identified two endothelial cell subtypes, aEC_cluster and EC2_cluster, which displayed potential associations with risk loci for large atherosclerotic stroke. Our GSEA enrichment analysis further uncovered abnormalities in pathways related to fluid shear stress and smooth muscle development in these two cell subtypes, with key genes such as Fos and Nr4a1 playing potentially critical roles. Pseudotime analysis provided insights into the differentiation stages of these two potentially critical endothelial cell types, with aEC_cluster representing the terminal stage of differentiation where the expression of Fos and Nr4a1 gradually increased over proposed time. Additionally, our transcriptional regulatory network analysis revealed the presence of regulon modules closely related to aEC_cluster, in which Gata2 and Irf7 extensively regulated the molecular network involving Fos, Nr4a1, and other significant alterations in the aEC_cluster. Collectively, our results suggest that these two endothelial cell subtypes play a potentially critical role in the pathogenesis of large atherosclerotic stroke and that the identified genes and pathways could be potential therapeutic targets for the treatment of stroke.

Under physiological conditions, endothelial cells play a crucial role in maintaining a dynamic balance between anticoagulation and fibrinolysis, and in vascular injury and repair (Krüger-Genge et al., 2019). Conversely, endothelial cell dysfunction is closely linked to cerebral small vessel disease, atherosclerosis, and stroke (Bai et al., 2022). For example, Wang et al. demonstrated that enhanced autophagy in endothelial cells contribute to increased formation of atherosclerosis (Wang et al., 2024). In our study, we investigated the genetic basis of single-cell expression profiles of endothelial cells in relation to stroke GWAS risk loci, and identified aEC_cluster and EC2_cluster as possible pathogenic cell subtypes in large artery atherosclerotic stroke. We found that the expression profile of aEC cells was significantly upregulated in pathways related to fluid shear stress, atherosclerosis, and TNF signaling pathway, while EC2_cluster showed downregulation in the muscle tissue development pathway. Both atherosclerosis and inflammation play crucial roles in the development of large artery atherosclerotic stroke (Jiang et al., 2022; Acosta et al., 2019). In addition, vascular smooth muscle is a crucial component of the vasculature, and an imbalance in its homeostasis can lead to the formation of atherosclerotic plaques (Grootaert et al., 2021). Therefore, blood flow shear factors may contribute to the shift of endothelial cells toward a stroke-causing subtype, leading to impaired regulation of smooth muscle cell homeostasis, atherosclerosis formation, and ultimately large-artery atherosclerotic stroke.

Among the differential genes significantly altered in the aEC_cluster and EC2_cluster, Fos plays a central role in pathways related to fluid shear stress, atherosclerosis, and muscle tissue development. A previous study suggests that Fos plays a critical role in the development of atherosclerosis (Miao et al., 2022) and it has been demonstrated that TNFα can upregulate Fos levels in vascular lesions (Goetze et al., 2001). In our study, we found significant upregulation of the TNF signaling pathway in the aEC_cluster (Figure 3), which partially supports a role for Fos in aEC_cluster-ssociated atherosclerotic stroke.

Transcription factors play a crucial role in cell differentiation processes (Stadhouders et al., 2019). In this study, we investigated the transcription factors involved in the differentiation of endothelial cells into pathogenic subtypes by analyzing the transcriptional regulatory network. Our analysis revealed that the activities of Gata2, Irf7, and Jund were significantly elevated in the terminally differentiated aEC_cluster (Figure 5). Specifically, Gata2 and Jund together regulate the expression of Fos. GATA2 is a member of the GATA binding factors family that regulates the expression of multiple genes by interacting with other DNA motifs and transcription factors (Rodrigues et al., 2012). Previous research has shown that GATA2 can promote the progression of atherosclerosis by upregulating NRP2 (Luo et al., 2022; Yin et al., 2020). Jund, a member of the Jun family, forms the activated protein-1 complex (AP-1) by heterodimerizing with members of the Fos family, which plays a vital role in cell migration, proliferation, and apoptosis (Mechta-Grigoriou et al., 2001; Eferl and Wagner, 2003). Therefore, the upregulation of Fos through the actions of Gata2 and Jund may have mediated the differentiation of endothelial cells into the critical aEC_cluster, therefore impacting the development of large atherosclerotic strokes. Additionally, our analysis identified numerous targets of Irf7 regulation in the aEC_cluster. Previous studies have demonstrated that Irf7 can exacerbate macrophage inflammation and disrupt cholesterol metabolism, thereby promoting atherogenesis (Senatus et al., 2020). Therefore, Irf7 may represent a potential target for promoting the differentiation of endothelial cells into pathogenic subtypes.

Despite the valuable insights provided by our study, several limitations should be acknowledged. First, scRNA-seq data used for transcriptomic and regulatory analyzes were derived from mouse brain vasculature, which may not fully capture the complexity or exact cellular phenotypes present in the human cerebrovascular system. While murine models offer high-resolution cellular profiling and conserved vascular architecture, species-specific differences in gene expression, transcriptional regulation, and disease susceptibility may limit the direct translatability of our findings. While several single-cell datasets exist for the cerebral vasculature, most are based on drop-seq technology, which has a significantly lower number of detected genes compared to the single-cell dataset obtained through SMART-seq2 (Ziegenhain et al., 2017; Svensson et al., 2018). Therefore, for the purpose of assessing the heritability of stroke risk loci, a higher number of genes detected is more favorable for inference. Hence, we selected the single-cell dataset for the cerebral vasculature obtained on the SMART-seq2 platform. Future studies incorporating human-derived single-cell or single-nucleus RNA-seq datasets, particularly from postmortem stroke patient samples or iPSC-derived vascular models, will be essential to validate and refine the identified endothelial subtypes and regulatory networks.

In addition, although transcription factors such as GATA2 and IRF7 were identified as potential key upstream regulators of pathogenic endothelial differentiation, further animal experiments are needed to validate this hypothesis. Moreover, direct pharmacological targeting of transcription factors remains challenging due to their intracellular localization and lack of defined binding pockets. Nonetheless, emerging therapeutic strategies—including antisense oligonucleotides (ASOs), RNA interference (RNAi), and CRISPR-based gene regulation tools—offer promising avenues to modulate transcription factor activity with higher specificity (Adachi et al., 2021). For example, ASO-based drugs have already shown clinical success in other neurological and cardiovascular conditions, suggesting that RNA-based therapeutics could provide feasible intervention strategies for modulating GATA2/IRF7-driven gene networks in stroke (Nitschke and Minassian, 2024; Dhuri et al., 2020).

In summary, our study highlights the significance of two potentially critical endothelial cell subtypes in the pathogenesis of large atherosclerotic stroke and identifies potential therapeutic targets. Gata2 and Irf7 emerge as theoretically critical upstream transcriptional regulators promoting endothelial cell differentiation toward critical subtypes, highlighting their potential as intervention targets for the prevention of large atherosclerotic stroke.

## Data Availability

The original contributions presented in the study are included in the article/Supplementary material, further inquiries can be directed to the corresponding author.

## Footnotes

^1^ https://gwas.Mrcieu.ac.uk/

## Supplementary material

SUPPLEMENTARY FIGURE 1 Ranking of regulon scores across different endothelial cells. Each point in the scatter plot represents a cell type.

SUPPLEMENTARY TABLE 1 Transcription factors and their associated modules.

SUPPLEMENTARY TABLE 2 Regulon activity scores (AUC) across cell types.
