# Supplementary figures and images for: Integrating scRNA-seq and GWAS data reveals potentially critical endothelial cells in large artery atherosclerotic stroke
Source: Front Neurosci. 2025 Sep 2;19:1646993. doi: 10.3389/fnins.2025.1646993 (PMC12436452; doi:10.3389/fnins.2025.1646993)

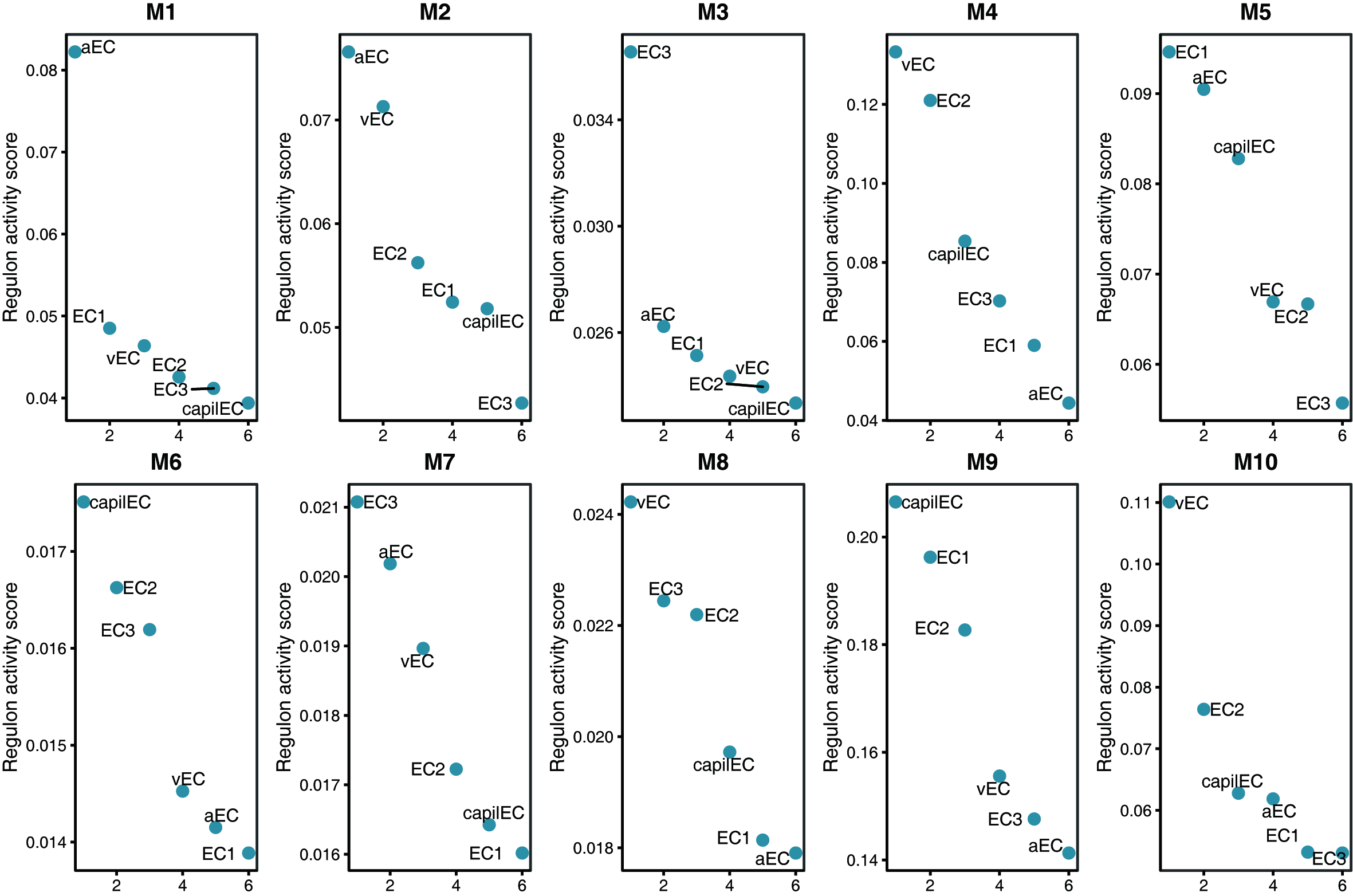

Supplement: SUPPLEMENTARY FIGURE 1 — Ranking of regulon scores across different endothelial cells. Each point in the scatter plot represents a cell type. [file Image_1.TIF]
